# Classic Hairy Cell Leukemia and Related Disorders: An Updated Review of Molecular Features and Personalized Therapies

**DOI:** 10.3390/jpm16060276

**Published:** 2026-05-22

**Authors:** Pratik Vijay Shah, Anita Ng, Andrew Shih, Tony Pham, Justin Wang, Houman Khalili, Cho Han Chiang, Adit Singhal, Alix Rosenberg, Sally Ko, Monica Wallin, Douglas E. Gladstone

**Affiliations:** 1 Department of Hematology/Oncology, Northwell Health Cancer Institute, New Hyde Park, NY 11042, USAasinghal2@northwell.edu (A.S.); dgladstone@northwell.edu (D.E.G.); 2 Department of Experimental Immunology, Feinstein Institutes for Medical Research, Manhasset, NY 11030, USAtpham5@northwell.edu (T.P.);; 3 Department of Computational Genomics, Feinstein Institutes for Medical Research, Manhasset, NY 11030, USA; 4 Donald and Barbara Zucker School of Medicine at Hofstra/Northwell, 500 Hofstra University, Hempstead, NY 11549, USA

**Keywords:** Hairy Cell Leukemia, Hairy Cell Variant

## Abstract

Classic Hairy Cell Leukemia (cHCL) and related conditions are rare, indolent B-cell malignancies characterized by distinctive morphological, immunophenotypic, and molecular features. Over the past decade, major advances in understanding the pathophysiology and molecular underpinnings have reshaped diagnostic and therapeutic approaches. This review synthesizes current knowledge on the cellular origins and signaling pathways that drive cHCL and Hairy Cell Variant (HCL-v)/splenic B-cell lymphoma/leukemia (SBLPN) and other molecular aberrations in disease pathogenesis. We discuss evolving diagnostic modalities, including flow cytometry, immunohistochemistry, and next-generation sequencing, that enhance diagnostic precision and disease monitoring. Additionally, we examine established and emerging therapeutic strategies—from purine nucleoside analogs (PNA) to targeted inhibitors and immunotherapies—that have significantly improved patient outcomes while highlighting challenges such as relapse and treatment resistance. By integrating insights from molecular biology and clinical practice, this review aims to provide a comprehensive understanding of cHCL and related disorders.

## 1. Introduction

Classic Hairy Cell Leukemia (cHCL) is a rare hematologic malignancy representing about 2% of new lymphoid leukemias [1]. An analysis from 1978 to 2004 identified 2856 cHCL cases, reflecting an incidence rate of 0.32 per 100,000 person-years with a nearly four-fold greater incidence among men compared to women and a greater than three-fold higher incidence in Caucasian patients compared to African American patients. The median age of presentation was 50 years, with a 10-year survival rate of >90% [2]. The WHO-HAEM5 classifies cHCL in the splenic B-cell lymphoma and leukemia family, which now also includes splenic B-cell lymphoma/leukemia with prominent nucleoli (SBLPN), splenic diffuse red pulp small B-cell lymphoma (SDRPL) and splenic marginal zone lymphoma (SMZL) as distinct entities [3]. The Hairy Cell Variant, previously recognized in the WHO-HAEM4R, is no longer an entity in the WHO-HAEM5 and has been replaced by SBLPN. SBLPN was created to absorb cases previously diagnosed as HCL variants as well as other splenic B-cell lymphomas with overlapping morphological and immunophenotypic features. Interestingly, the ICC classification continues to recognize the HCL variant [4].

In comparison to cHCL, SBLPN is exceedingly rare, accounting for 0.4% of chronic lymphoid malignancies with an incidence of 0.03 per 100,000 persons per year [5] and more frequently affecting the elderly, with a male predominance. SDRPL is also extremely rare, affecting older adults with a slight male predominance. SMZL accounts for <2% of all lymphoid malignancies with a median age of diagnosis of 69 years and an incidence of 0.13 per 100,000 persons per year and demonstrates a predilection for males and individuals of Caucasian descent [6].

This review provides updated knowledge regarding classic HCL (cHCL) and the HCL-like disorders SBLPN and SDRPL.

## 2. Molecular Features of Classic HCL

Classic HCL is defined as a mature B-cell leukemia. Most cases (90–95%) are associated with a *BRAF V600E* somatic mutation [3]. This mutation leads to the transversion of a thymine (T) with an adenine (A) in exon 15 of *BRAF* at position 1799 of the gene-coding sequence located in chromosome 7q34, leading to a valine (V) being replaced by glutamate (E) at position 600 [7]. In these tumors, downstream activation of MEK and ERK proteins leads to constitutive activation of proliferation and pro-growth signals. The *BRAF V600E* mutation is considered to be the driver mutation in cHCL.

Importantly, 5–10% of patients with cHCL do not harbor the *BRAF V600E* somatic mutation. These cases may harbor alternative *BRAF* mutations, including exon 11 F468C and D449E [8,9]. Rare cases are *BRAF* wild type; these patients may present with mutations in the *KLF2* (transcription factor) or the *CDKN1B/p27* (cell cycle inhibitor) genes. Some of these genetic alterations occur at sites targeted by activation-induced cytidine deaminase (AID), suggesting AID-mediated mutagenesis as the mechanism of acquiring these somatic mutations in *BRAF* wild type cHCL [10]. Of note, mutations of *TP53* occur in cHCL at a lower frequency than other lymphoid malignancies [11].

## 3. Molecular Features of SBLPN

In SBLPN, *BRAF* is wild type; however, there is an enrichment of *MAP2K1/MEK1* mutations along with mutations in epigenetic regulator genes (*KDM6A*, *EZH2*, *CREBBP*, *ARID1A*) [10]. Methylation profiling identifies four molecular clusters in SBLPN, with the first cluster mainly consisting of *MAP2K1* (91%), *CREBBP*, *ARIDIA*, and *TERT* promoter mutations [12]. The other clusters had lower rates of *MAP2K1* mutations, while *TP53* mutations were seen in 20–50%. These clusters provide important observations of the heterogeneity of mutations seen in patients with SBLPN.

It is important to appreciate that both *BRAF* mutations and *MAP2K1* mutations activate the same MAPK (RAF-MEK-ERK) pathway in these diseases; however, *BRAF V600E* is the canonical upstream driver of cHCL, whereas *MAP2K1/MEK1* mutations are downstream pathway mutations seen mainly in SBLPN [9].

## 4. Molecular Features of Other Splenic B-Cell Lymphoma/Leukemias

SDRPL expresses various somatic mutations, with missense activating mutations in *CCND3* present in the majority of these patients [4,13]. A hemizygous 7q deletion is identified in approximately one-third of SMZL cases. This finding is rare among lymphoid malignancies, and may occasionally be confused with a del (7q) myelodysplastic syndrome given the similar clinical presentation of splenomegaly with cytopenias. Gene expression studies reveal that the most frequently mutated genes in SMZL include *nuclear factor κB* (*NF-κB*), *NOTCH2*, and *KLF2* [14].

## 5. Diagnostic Criteria and Differential Diagnosis

The common symptomatic presentations of cHCL are fatigue, early satiety and recurrent infections. The clinical hallmark is splenomegaly without significant lymphadenopathy (Table 1). Complete blood counts commonly reveal neutropenia or pancytopenia with an absolute monocytopenia [14]. Leukemic cells may be difficult to identify on a peripheral blood smear; however, when present, B lymphocytes are medium in size with blue cytoplasm and serrated borders. B lymphocyte immunophenotyping is required to establish a cHCL diagnosis and to help distinguish cHCL from SBLPN, SDRPL and SMZL. The cHCL B lymphocytes co-express CD19^+^, CD20^+^, CD11c^+^, CD25^+^, CD103^+^, CD123^+^, CD72^+^ (DBA), and Annexin-1^+^ with intense staining for CD200 expression and negative staining for CD27 antigen [4,14]. A bone marrow biopsy and aspiration at diagnosis allow for determination of extent of infiltration, fibrosis, and cellularity of the marrow. Immunohistochemical (IHC) stains for Tartrate-resistant acid phosphatase (TRAP), VE1 (BRAFV600E) or PD-1 could aid in diagnosis as well [15].

Patients diagnosed with SBLPN present with similar symptoms as for cHCL. On laboratory evaluation, monocytopenia is rare and lymphocytosis is common. A peripheral blood smear reveals medium to large B lymphocytes with poorly defined cytoplasmic projections with a large prominent single nucleolus. Flow cytometry reveals clonal cells that express CD20, CD11c, and CD103, but in contrast to cHCL, the patients with SBLPN have B lymphocytes that are CD25(dim) or CD25^−^, Annexin-1^−^ and CD123^−^. IHC for TRAP is usually negative, and BRAFV600E IHC, polymerase chain reaction (PCR), and next-generation sequencing (NGS) testing is expected to be negative. Significant reticulin fibrosis is rare upon marrow evaluation [5].

SDRPL presents with splenomegaly and cytopenia. A CD200 mean fluorescence intensity (MFI)/CD180 of <0.5 on flow cytometry favors a diagnosis of SDRPL over HCL, SMZL and SBLPN. Histologically, bone marrow examination shows a predominant intrasinusoidal pattern of involvement which is in contrast to cHCL, which shows a diffuse pattern with reticulin fibrosis [3].

SMZL presents with splenomegaly and cytopenia. Patients with SMZL can also present with an autoimmune manifestation [16]. On peripheral blood flow cytometry, SMZL B lymphocytes consistently express CD20, CD79a, BCL2, and surface immunoglobulin while negative for CD5, CD10, BCL6, cyclin D1, annexin A1, CD103, and CD123. On immunohistochemistry, TRAP is negative. Histologically, SMZL develops in the white pulp of the spleen with medium-sized B cells organized into a pale ring around the follicle with a marginal zone pattern [17].

**Table 1:** Pathologic features of B cell neoplasms.

	Classic Hairy Cell Leukemia	Splenic B-Cell Lymphoma with Prominent Nucleoli (SBLPN)	Splenic Diffuse Red Pulp Lymphoma (SDRPL)	Splenic Marginal Zone Lymphoma (SMZL)
Typical presentation	Middle-aged, male predominance	Older adults, no strong sex bias	Older adults, slight male predominance	Older adults, often >50 years
Peripheral blood cells morphology	Small- to medium-sized B lymphocytes, circumferential “hairy” projections	Larger cells with prominent nucleoli, irregular or polar cytoplasmic projections	Small monomorphic B cells with villous cytoplasm	Small- to medium-sized B cells; “villous B lymphocytes” with polar cytoplasmic projections
Blood count pattern	Pancytopenia frequent; monocytopenia and neutropenia characteristic	Anemia and thrombocytopenia common; leukocytosis rather than cytopenias	Variable cytopenias	Moderate lymphocytosis
Spleen involvement pattern	Predominantly red pulp, diffuse infiltration; white pulp often atrophic	Predominantly red pulp	Purely or predominantly diffuse red pulp infiltration, with effacement of white pulp	Predominantly white pulp expansion
Bone marrow features	“Fried-egg” cells and marked reticulin fibrosis; aspiration often “dry tap”	Infiltration without marked fibrosis	Involvement often diffuse but fibrosis is limited	Marrow involvement common with nodular, interstitial or intrasinusoidal pattern
Key immunophenotype (flow/IHC)	Bright surface Ig (usually IgG), CD19^+^, CD20 bright, CD22^+^, CD11c^+^, CD25^+^, CD103^+^, CD123 bright^+^, annexin A1^+^, TRAP^+^	Bright surface Ig (often IgG), CD19^+^, CD20^+^, CD22^+^, CD11c^+^, CD103^+^, but CD25^−^, CD123^−^, annexin A1^−^, TRAP^−^	CD19+, CD20+, CD22+, often CD11c+, usually CD25^−^, CD103^−^, CD123^−^, annexin A1^−^, TRAP^−^;	CD19+, CD20+, surface IgM/IgD+, typically CD5^−^, CD10^−^, CD23^−^, CD43^−^, CD103^−^, annexin A1^−^, cyclin D1^−^; characteristic “targetoid”
Genetics (common)	*BRAF V600E* mutation in vast majority	*MAP2K1* mutations frequent subset	Recurrent mutations in *CCND3* and others described	Frequent 7q deletion

## 6. Risk Stratification and Prognostic Factors

There are specific treatment criteria in cHCL; however, there are no standardized risk stratification guidelines to help clinicians prognosticate which patients are at higher risk of progression to requiring treatment. Patients with bulky splenomegaly (>10 cm below costal margin), higher beta-2 microglobulin, leukocytosis (>10 × 10^9^/L) and an elevated hairy cell count are deemed to have a higher tumor burden and may be at higher risk of requiring immediate treatment [1]. Retrospective data suggests that elevated lactate dehydrogenase (LDH) levels at the time of diagnosis may be predictive of relapse/progression risk [18].

Sex may also have an impact on outcomes in cHCL. A data registry analysis showed longer median time to next treatment in females compared to males, which held true after adjusting for response rates and BRAF status [19].

Molecular features, particularly immunoglobulin heavy chain variable region mutational status and gene usage (*IGHV* mutational status), are recognized as key biological and prognostic markers in cHCL [14]. It is important to obtain mutational status at time of diagnosis in patients with cHCL, SBLPN, SDRPL, and SMZL. The vast majority of cHCL cases express *IGHV* sequences that are mutated, defined as <98% sequence identity with the germline sequence [20]. In contrast, a high proportion of patients with SBLPN will be unmutated at diagnosis. Prognostically unmutated patients present with bulkier disease, require treatment faster, and are less responsive to treatment than mutated patients [20].

VH4-34 expressing leukemia cells represent a distinct subgroup associated with poor prognosis and suboptimal responses. VH4-34 cases were more frequently seen in patients with SBLPN (40%) and SMZL (20%) compared to cHCL (10%) [21]. The VH4-34 heavy chain sequence is frequently unmutated (40%) compared to non-VH4-34 cases (10%). These patients often present with a higher white blood cell count at diagnosis. Patients with the VH4-34 rearrangement were seen to have lower response rates to purine nucleoside analog (PNA) therapy and worse progression-free survival after initial therapy. This rearrangement is predictive of a poor response to first treatment and overall outcome independent of patient’s diagnosis (cHCL or SBLPN) [22]. Interestingly, VH4-34 expressing B cells in other lymphoproliferative conditions have also had strong associations with autoimmune phenomena, highlighting the intrinsic autoreactive potential of these antibodies [21].

NGS assessment is critical when engaging in prognostic discussions. As described earlier, *BRAF*, *MAP2K1*, and *TP53* mutations all have various treatment implications; *BRAF* mutants often enjoy a good first response to PNA and targeted treatments in the relapsed setting, whereas *MAP2K1* and *TP53* mutants are PNA-refractory and do not have effective targeted treatments in the refractory setting. *MAP2K1* mutations are seen in clones that have *IGHV* 4-34 usage [23].

## 7. Current Treatment Paradigms for Classic HCL

### 7.1. Treatment Indications

Based on updated 2026 guidelines, treatment for cHCL is indicated for constitutional symptoms, symptomatic splenomegaly, and cytopenias (Hgb < 10.0 g/dL, neutrophil count < 1 × 10^9^/L, platelet count < 100 × 10^9^/L)^14^. It is becoming established that disease velocity is a more critical determinant of medical intervention than strict cell counts, as cHCL has a lower proliferation rate than most leukemias; therefore, monitoring of cytopenias and symptoms can be continued for an extended time before initiation of therapy.

### 7.2. Approved Frontline Purine Nucleoside Analog (PNA) Monotherapy

Treatment for cHCL is not curative in intent but rather aims to achieve prolonged disease control. Initial treatment for HCL includes PNA monotherapy. There are several accepted induction therapies for cHCL, including single agent PNA therapy, PNA therapy followed by monoclonal antibody therapy, concurrent PNA therapy with monoclonal antibody therapy and concurrent BRAF-directed therapy with monoclonal antibody therapy.

The two most effective PNAs are pentostatin (2′-deoxycoformycin; dCF) and cladribine (2-chlorodeoxyadenosine; CdA). Pentostatin’s mechanism of action is via the inhibition of adenosine deaminase, which results in an accumulation of deoxyadenosine and adenosine in B lymphocytes. Accumulated deoxyadenosine is phosphorylated to deoxyadenosine triphosphate (dATP), which induces DNA strand breaks and leads to the activation of p53 and release of cytochrome c from mitochondria and apoptosis [24]. Cladribine produces a distinct metabolic intermediate that ultimately yields a similar outcome, inducing DNA strand breaks and apoptosis of malignant B lymphocytes.

As early as the 1980s, the effectiveness of monotherapy PNA in cHCL was established with complete response (CR) rates ranging from 70 to 90% and the duration of hematologic response usually lasting more than 10 years [25,26,27,28,29,30,31,32,33,34,35,36,37,38]. Hematologic adverse effects of pentostatin and cladribine include myelosuppression and significant T-lymphocyte suppression [39]. Additionally, both agents can induce rashes and peripheral neuropathy, which can in some cases be progressive and permanent [40]. There have been no direct comparisons of pentostatin to cladribine. Cladribine can be administered intravenously or subcutaneously daily for five days, while pentostatin is administered intravenously every 14 days. Globally, this approach has been utilized and responses hold up throughout different population types [41].

### 7.3. Frontline Concurrent Chemoimmunotherapy

Classic HCL is characterized by high-density expression of CD20 on malignant B lymphocytes. This elevated antigen expression facilitates efficient binding of anti-CD20 monoclonal antibodies, promoting immune-mediated recognition and elimination of malignant cells. Furthermore, concomitant administration of PNA and anti-CD20 monoclonal antibody is hypothesized to enhance therapeutic sensitivity, potentially increasing the susceptibility of malignant B lymphocytes to PNA-induced cytotoxicity. This synergistic interaction supports the rationale for early incorporation of anti-CD20-directed therapy in the treatment of cHCL. When studied versus PNA monotherapy, concurrent combination therapy had a 100% CR rate at 6 months with a 97% undetectable measurable residual disease (uMRD) rate vs. an 88% CR rate and 24% uMRD rate for monotherapy [42,43].

### 7.4. Frontline Sequential Chemoimmunotherapy

Combination chemoimmunotherapy sequentially has also been studied in the frontline setting. Sequential PNA treatment followed by monoclonal anti-CD20 antibody immunotherapy (rituximab, obinutuzumab) results in high CR rates, and in a small retrospective observational study it showed improved disease-free survival compared to PNA monotherapy [44]. Sequential therapy of cladribine followed by rituximab has been studied with cladribine administered first over five days followed by eight weekly doses of rituximab given at standard doses [45].

### 7.5. Combination Versus Monotherapy Approach

Both combination chemoimmunotherapy and PNA monotherapy are approved in the front line setting for patients with indication to treat for cHCL. Choosing between these approaches requires a nuanced approach. Historically, single agent PNA has yielded high CR rates with durable responses lasting a decade or more in many patients. However, it has become increasingly recognized that a monotherapy approach may not offer the deepest of responses. In patients who desire the deepest possible remission with the goal of long-term disease-free survival, combination approaches with sequential or concurrent chemoimmunotherapy may be considered. These approaches are reasonable to consider in younger and fitter patients who may have >15–20 years of life remaining. The addition of monoclonal anti-CD-20 therapy provides higher uMRD and CR rates; however, this benefit should be balanced with the greater rates of cytopenias (thrombocytopenia), which may result in increased toxicity. An approach to mitigate the toxicities of combination therapies can be to sequentially deliver them: the anti-CD20 monoclonal antibody is administered after the completion of PNA to help mitigate some of the immunosuppressive burden while still achieving an improved depth of response compared to PNA alone. For patients who are older, more frail, or have significant infectious risk concerns, PNA monotherapy remains a highly effective and reasonable choice, with the anti-CD20 monoclonal antibody reserved for consolidation sequentially if residual disease is detected or deferred entirely until relapse.

Risk factors such as presence of adverse molecular features which may portend an inferior response to PNA monotherapy may argue more for the use of a combination approach or even alternative strategies, as discussed in the following sections.

Febrile neutropenia is a serious and common complication of cHCL treatment, particularly with PNA (cladribine), leading to a reported 3% mortality rate [46]. Classic HCL patients are often neutropenic at presentation. In such scenarios, delaying or avoiding using PNA therapies in this setting is appropriate and the use of an anti-CD20 therapy with a BRAF inhibitor plus a granulocyte growth factor should be considered.

## 8. Management of Relapsed/Refractory (R/R) Classic HCL

### 8.1. Approved R/R Therapeutic Options

Patients treated with PNA monotherapy relapse at a rate of 30–40% at 10–12 years [47]. The vast majority of these patients require retreatment. If disease relapse occurs after 24 months of initial PNA therapy, rechallenging with either PNA monotherapy or concurrent therapy is acceptable [14]. For a relapse occurring within 24 months, it is suggested to confirm the diagnosis with repeat morphological and molecular evaluation of the bone marrow [14].

The most utilized targeted interventions are the oral BRAF inhibitors, namely Vemurafenib and Dabrafenib. Vemurafenib, an oral BRAF inhibitor approved for relapsed cHCL after PNA treatment, results in CR rates ranging between 35 and 42% [48]. Additionally, in R/R HCL, re-treatment with Vemurafenib has also been shown to be safe and effective [49]. Vemurafenib is also attractive as a time-limited therapy. Common adverse effects include a maculopapular rash, arthralgias, photosensitivity, pruritus, fatigue, alopecia, pyrexia, and pancreatitis. Prolonged exposure is associated with non-melanotic cutaneous malignancies.

Approved for use in the United States in patients with R/R cHCL after ≥2 prior systemic therapies including a PNA is Moxetumomab Pasudotox (Moxe). Moxe is a fusion of an anti-CD22 fragment with pseudomonas exotoxin. After binding CD22, which is highly expressed in HCL cells, it is internalized and the toxin blocks protein synthesis leading to apoptotic death of these cells [50]. CD22 is an ideal target in HCL as it is strongly and consistently expressed in HCL with rapid internalization and no recycling [51].

Phase I testing for Moxe achieved a CR rate of 57% in patients with R/R HCL with durable CRs based on uMRD at 5 years [52]. A pivotal phase III trial reported ORR of 75%, CR rate of 41%, and 85% uMRD rate in responders. Moxe was administered intravenously for ≤6 cycles [53]. Common adverse effects include peripheral edema, nausea, fatigue, headache, and transaminase rise. Serious but usually reversible adverse effects include hemolytic uremic syndrome (HUS) and capillary leak syndrome (CLS), seen in about 5–10% of patients, which is usually managed with hydration, monitoring, and treatment interruption. There is less prolonged myelo- and immunosuppression than PNA, with T lymphocytes largely spared [53].

Interferon-alpha, although approved, is very rarely utilized in the R/R setting and may be considered in patients who are pregnant and require treatment.

### 8.2. Off-Label Combination Approaches for R/R Treatment

Bone marrow biopsies of patients after use of single-agent Vemurafenib in CR at 16–18 weeks commonly revealed 5–10% residual CD20-expressing leukemic cells. This was the rationale for the combination of Vemurafenib with anti-CD20 therapy [54]. A small phase 2 trial of Vemurafenib followed by Rituximab showed a CR rate of 87% with a 65% uMRD rate.

Despite this combination, in vitro studies still showed persistent ERK phosphorylation (pERK+) despite ongoing BRAF inhibition, suggesting bypass MEK/ERK downstream activation of BRAF as a resistance mechanism. This was the rationale for developing a dual BRAF and MEK inhibition strategy. Strategies include dabrafenib + trametinib or vemurafenib + cobimetinib.

Dabrafenib, a reversible BRAF inhibitor which more selectively targets the BRAF kinase than vemurafenib, has also been studied as a time-limited monotherapy in frontline and R/R HCL and in combination indefinitely with trametinib, an MEK inhibitor. Studies show acceptable overall response rates and complete response rates in the relapsed, heavily pre-treated setting. Adverse effects included higher rates of non-melanoma skin cancers [55,56].

Adding rituximab to Moxe increases MRD-negative CR rates and also prevents or delays the development of anti-drug antibodies that develop against Moxe by eradicating normal B cells that are responsible for the production of these antibodies. Moxe in combination with rituximab can also be an off-label approach to consider to more quickly reduce HCL burden and quicken uMRD CR [57].

## 9. Management of SBLPN

Concurrent cladribine and rituximab is currently the standard of care as an initial treatment for SBLPN. Interestingly, pivotal combination studies utilized an MRD-guided approach to treatment with re-treatment at six months with rituximab for patients with MRD positivity after initial combination chemoimmunotherapy. These patients demonstrated a sustained response with high 5-year PFS, OS, and uMRD rates [58,59].

## 10. Other Novel Investigational Options

BTK inhibitors (BTKi) are commonly used in other B cell malignancies. Small trials using both first- and second-generation BTKi have revealed modest overall response rates in the frontline and relapsed setting [60,61].

BCL2 inhibitors have shown activity in vitro against HCL [62]. Venetoclax was studied in a cohort of six patients with R/R HCL in a time-limited fashion, followed by sequential addition of rituximab in half the cohort which demonstrated activity [63]. This has led to a phase II trial that is currently studying venetoclax in the treatment of patients with R/R HCL [64].

Receptor tyrosine kinase-like orphan receptors 1 and 2 (ROR1 and ROR2) have also been found to be overexpressed on HCL cells. These receptors function as crucial cell surface receptors for Wnt signaling, which regulates cell migration, proliferation, and differentiation. Primarily active during embryogenesis, they are overexpressed in B cell malignancies and have been shown to promote B-cell survival and proliferation. Monoclonal antibodies targeting ROR1- and ROR2-dependent signaling are being studied and have shown promising results [65].

Cellular therapies including CAR-T cells are also being considered for use in HCL-v/SBLPN. One such CAR-T construct is a product which targets the IGVH4-34 BcR specifically. The goal is that this CAR-T product will specifically target pathogenic B cells in various malignancies, including cHCL/SBLPN [66]. Preclinical work on a CAR-T product that targets the three receptors of the B cell activating factor (BAFF) has also been promising. BAFF is a type II transmembrane protein that activates NF-κB and related pathways upregulating anti-apoptotic Bcl-2 family proteins and supports B cell survival and function [67]. Preclinical findings of this CAR-T product have shown promise in the R/R HCL setting.

A study evaluating the safety and use of anti-CD22 CAR-T, which has been utilized in B-ALL, shows encouraging results in the R/R setting [68].

## 11. Role of Minimal Residual Disease Testing

Minimal residual disease (MRD) testing in HCL remains heterogeneous and without clear consensus on when its use is most appropriate [69]. MRD assessment in HCL trials has involved bone marrow IHC evaluation, multi-parameter flow cytometry (MPC) or PCR for detecting mutant *BRAF*. In cHCL, MRD is predictive of relapse risk [70]. In SBLPN, an undetectable MRD post-treatment at 4 weeks and 6 months appears to be critical with regard to PFS and OS benefits [59]. However, it is important to note that, after PNA monotherapy, there are cHCL patients who have remained in clinical hematologic remission for >15 years despite having bone marrow evidence of MRD [71]. Consensus guidelines from international experts in 2022 suggest that MRD assessment in HCL may be more critical in the relapse setting given that CR duration using established regimens is generally limited in this setting and MRD status can be an indicator of efficacy of the salvage regimen and provide useful information about the long-term efficacy of these therapies [70].

## 12. Conclusions, Future Directions and Unmet Needs

For future considerations, it is important to continue to refine the biology of HCL and related conditions and learn more about the clonal evolution and microenvironment through single-cell, multi-omic, and spatial approaches. From a therapeutic perspective, optimizing the sequencing and combination of targeted agents will be essential as novel therapies emerge. The optimization of MRD assessment and integration into clinical practice and trials will be a key question to address for the future of HCL research and care. Evaluating quality of life, survivorship, and long-term toxicities of targeted approaches from a patient-centric perspective will be indispensable.

## Data Availability

No new data were created or analyzed in this study. Data sharing is not applicable to this article.
